# Increasing availability of palatable prey induces predator-dependence and increases predation on unpalatable prey

**DOI:** 10.1038/s41598-021-86080-x

**Published:** 2021-03-24

**Authors:** Thomas J. Hossie, Kevin Chan, Dennis L. Murray

**Affiliations:** Department of Biology, Trent University, 2140 East Bank Drive, Peterborough, ON K9J 7B8 USA

**Keywords:** Ecology, Behavioural ecology, Community ecology, Population dynamics

## Abstract

Understanding the factors governing predation remains a top priority in ecology. Using a dragonfly nymph-tadpole system, we experimentally varied predator density, prey density, and prey species ratio to investigate: (i) whether predator interference varies between prey types that differ in palatability, (ii) whether adding alternate prey influences the magnitude of predator interference, and (iii) whether patterns of prey selection vary according to the predictions of optimal diet theory. In single-prey foraging trials, predation of palatable leopard frog tadpoles was limited by prey availability and predator interference, whereas predation of unpalatable toad tadpoles was limited by handling time. Adding unpalatable prey did not affect the predator’s kill rate of palatable prey, but the presence of palatable prey increased the influence of predator density on the kill rate of unpalatable prey and reduced unpalatable prey handling time. Prey selection did not change with shifts in the relative abundance of prey types. Instead, predators selected easy-to-capture unpalatable prey at low total densities and harder-to-capture palatable prey at high densities. These results improve our understanding of generalist predation in communities with mobile prey, and illustrate that characteristics of the prey types involved govern the extent to which alternate prey influence the predator’s kill rate.

## Introduction

Natural systems vary considerably in the abundance of prey and predators, as well as in the diversity of available prey types. Understanding how these factors interact to influence predation within a community has been a longstanding priority of ecologists^1–3^. Key developments in this area include recognition of non-linearities in the relationship between prey density and a predator’s per capita kill rate (i.e., the functional response^4^), refinement of functional response models to account for predator-predator interactions that depress per capita kill rate (i.e., interference^5–7^), and optimal foraging theory which helped explain prey selection based on prey availability and the relative profitability of alternate prey^3,8^. In each case, subsequent research has revealed additional complexities in how prey density, predator density, and availability of alternate prey interact to alter the realized patterns of predation. For example, ecologists now recognise that the availability of alternate prey can significantly influence the shape of the functional response^9–11^, as well as the magnitude of predator interference (e.g.^12^). At the same time, optimal diet models have performed poorly in systems with mobile prey^13^ highlighting the need to better understand patterns of predation in natural systems.

Ecological communities are composed of prey species that differ in habitat use, activity level, vulnerability to predators, and profitability as prey items. Faced with such variation in their prey, many generalist predators flexibly adjust their foraging behaviour to maximize encounter and capture rates (e.g.^10,14,15^). A classic example of this is the shifting of predator foraging habitat with changes in the relative availability of prey that occupy distinct habitats (i.e., frequency-dependent switching^9,14^). Frequency-dependent switching generates distinct patterns in indices of selective predation as well as in the predator’s functional response (i.e., a sigmoidal shaped response reflecting a region of density-dependent predation) and has been linked to increased stability in multi-prey communities (e.g.^16^; but see^17–19^). Another way predators adjust their foraging behaviour is through shifts in the amount of active prey search with changes in total prey density (e.g.^20,21^), which can similarly influence the functional response shape^22^. Predators may also change their foraging mode to match the behaviour of the prey species they are targeting. For example, active search can increase encounter rate with prey that are relatively inactive, but a ‘sit-and-wait’ strategy can reduce the detectability of predators by prey and is energetically efficient when hunting active prey (e.g.^15^). Critically, shifts in foraging strategy from ‘sit-and-wait’ to ‘active search’ come with a concomitant increase in encounter rate with other foraging predators, setting the stage for an increase in predator interference. In multi-prey communities the foraging mode that maximizes energetic gain should therefore depend on the density and behaviour of available prey items, as well as the density of conspecific (or heterospecific) predators.

It is now widely acknowledged that in the majority of predator–prey interactions a predator’s per capita kill rate tends to decline as predator density increases^23–25^. When both prey and predator density influence the prey consumption rate of an average predator, the functional response is said to be ‘predator-dependent’^24,25^. Predator-dependence can arise from a range of direct and indirect mechanisms including predator-predator conflict, group hunting, and depletion of easy-to-find prey^24,25^. As such, parameterization of functional response models is a widely used and reliable way to estimate the effect of interference on kill rates^22,23,25,26^. For example, some functional response models include a ‘mutual interference coefficient’ which reflects the degree to which attack rate declines with increases in predator density^22,23,25^. Yet, while predator-dependence has been the focus of intense study for more than 60 years (reviewed by^27^), studies experimentally examining the effect of predator density on the functional response remain few in part due to logistical and statistical constraints^28^. These investigations however continue to demonstrate strong impacts of predator density on the rate of prey consumption and have uncovered additional complexity in the ways that variation in predator density influences predation (e.g.^12,29,30^). Notably, while it has been demonstrated that alternate prey can critically influence the extent of predator interference^12^, this seemingly important result has subsequently received limited attention.

Optimal diet theory has provided ecologists with tools to make quantitative predictions about whether a forager should attack a given prey item or pass it over in search of more profitable prey^3,8^. The model predicts that: (1) less profitable prey (i.e., prey yielding less energy per unit handling time) should be dropped from the diet as the abundance of more profitable prey types increases, (2) the decision to specialize should not depend on the availability of less profitable prey types, and (3) the switch between generalist and specialist strategies should follow a quantitative threshold rule^8,31,32^. Essentially, when profitable prey are common, foragers should spend their time searching for profitable prey instead of handling less profitable prey. Sih and Christensen^13^ show that optimal diet theory has been less effective in predicting the diet of foragers that attack mobile prey and propose two reasonable explanations. First, handling time is often quantified as manipulation time after capture and inappropriately ignores pre-capture pursuit times which can be significant for mobile prey^13^. Second, when prey are mobile, predator diet is more strongly determined by variation in vulnerability among prey than by active choice^13^. Interestingly, prey selectivity can also vary with the total prey available (i.e., ‘rank switching’^33^). One proposed explanation for this is that predators target easy-to-capture prey when overall prey density is low, but switch to targeting more profitable prey types at higher total prey densities^33,34^. In such cases, the pattern of lethal predation should be governed by prey vulnerability at low total prey density, but by predator preference when total prey density is high, irrespective of their relative abundances.

Herein we examine the effects of prey density, predator density, and availability of alternate prey on the pattern and magnitude of predation. Specifically, we sought to identify whether predator interference varies between prey types that differ in palatability, and whether adding alternate prey influences the magnitude of predator interference. In addition, we sought to determine whether patterns of prey selection varied according to the broad predictions of optimal diet theory in a system with two mobile prey types. Our experimental system employed the tadpoles of two anuran species as prey and late-instar Aeshnidae dragonfly nymphs as predators. Antagonistic interactions between dragonfly nymphs, as quantified directly through behavioural observations, are well documented (e.g.^30^). Our prey types included Northern leopard frog tadpoles (*Lithobates pipiens* [family: Ranidae]) as a palatable prey type with strong behavioural and morphological defences^35,36^, and American toad tadpoles (*Anaxyrus americanus* [family: Bufonidae]) as an unpalatable prey type with less pronounced behavioural and morphological defences^37,38^. Specifically, baseline activity level, which influences detectability to predatory dragonfly nymphs, is significantly higher in American toad tadpoles than in leopard frog tadpoles^37^. Strike success by *Anax* dragonfly nymphs is also reported to be higher on American toad tadpoles than on leopard frog tadpoles^39^. Bufonidae toad tadpoles are however chemically protected by bufadienolides and biogenic amines which are effective against a range of predators including dragonfly nymphs^37,40,41^. Chemical defences are thought to increase prey handling time by prolonging digestion or reducing motivation to search for the next prey item^42^ and can therefore render these prey less profitable (i.e., by lowering the net rate of energetic intake).

We predicted that predation on the palatable prey type would be more strongly influenced by predator interference, resulting from higher motivation to search for and capture these prey. Building from the expectations of optimal diet theory, we predicted that the inclusion of unpalatable prey would have a negligible effect on the per capita kill rate of palatable prey, but that inclusion of palatable prey would reduce the kill rate of unpalatable prey. We did not have a prior predictions about the shape of the functional responses, but did expect predator density to exhibit some influence on per capita kill rate given the aggressive nature of late instar dragonfly nymphs^30^. Finally, we predicted that prey selection would depend on the total prey density, following the general expectations rank switching.

## Results

### Profitability trials

To evaluate differences in detectability, catchability, and palatability between leopard frog and toad tadpoles, we presented tadpoles to dragonfly nymphs in small arenas and collected detailed behavioural data. Preliminary examination found no effect of prey sequence on our metrics, so we restricted analysis to the first prey item presented to each predator when hunger was better standardized across individuals. Leopard frog tadpoles took significantly longer to capture than toad tadpoles (*W* = 97, *P* = 0.04; Table 1), and were clearly palatable given that dragonfly nymphs completely consumed each of these prey items. In contrast, nymphs only consumed ~ 79% of toad tadpole carcasses (range = 21–100%), suggesting that they were significantly less palatable (*t*_17_ = − 2.77, *P* = 0.013; Table 1). Time to first strike, strike success, and time spent consuming the prey item did not differ significantly between prey types (all *P* > 0.47, Table 1). Prey profitability was similar between prey types (see Supplementary Material). We note that prey used in this experiment were larger than those used in the functional response experiment below, so estimates may not translate directly to that experiment.

**Table 1:** Descriptive statistics (mean ± SE) from behavioural observations made during staged predator–prey interactions between late-instar Aeshnidae dragonfly nymphs presented with an individual leopard frog or toad tadpole.

Prey	tStrike (*s*)	Strike Success	tCapture (*s*)	tConsume (*s*)	Proportion consumed
A. Leopard A. Leopard	235.11 ± 96.66 345.11 ± 235.30	0.70 ± 0.10 0.61 ± 0.13	486.77 ± 176.40 439.56 ± 230.67	505.33 ± 85.47 592.44 ± 101.96	1.00 ± 0.00 1.00 ± 0.00
B. Leopard B. Toad	319.11 ± 145.98 64.33 ± 22.14	0.60 ± 0.11 0.83 ± 0.08	408.67 ± 142.39 86.11 ± 25.59	545 ± 145.82 564.89 ± 147.34	1.00 ± 0.00 0.78 ± 0.11
C. Toad C. Leopard	68.33 ± 36.36 553.22 ± 197.29	0.74 ± 0.10 1.00 ± 0.00	106.67 ± 64.27 703.89 ± 316.36	428.11 ± 120.09 247.44 ± 74.62	0.82 ± 0.09 0.81 ± 0.09
D. Toad D. Toad	257 ± 114.77 238 ± 83.63	0.73 ± 0.11 0.87 ± 0.09	272.67 ± 111.32 264.78 ± 78.52	721.33 ± 229.42 655.78 ± 233.63	0.75 ± 0.13 0.75 ± 0.12
**Overall species-level averages using first prey item only**					
**Leopard**	277.11 ± 85.53	0.65 ± 0.07	447.72 ± 110.37	525.17 ± 130.54	1.00 ± 0.00
*Median* *Range*	81.00 2–1055	0.50 0.50–1.00	212.50 5–1401	395.50 142—1364	1.00 NA
**Toad**	162.67 ± 62.72	0.74 ± 0.07	189.67 ± 65.52	574.72 ± 82.13	0.79 ± 0.08
*Median* *Range*	46.00 1–94	1.00 0.25–1.00	66.00 1–974	514.00 7–2341	1.00 0.00–1.00

Trials took place in small circular arenas, and nymphs were presented with a second tadpole (of the same or different species) once they had finished consuming the first. Lettering in the Prey column demarks the four sequences offered to individual predators (*n* = 9 replicates per sequence).  tStrike = time to first strike at prey, Strike Success = proportion of successful strikes, tCapture = time to prey capture, tConsume = time spent feeding on captured prey item.

### Functional response experiment

**Figure 1:**
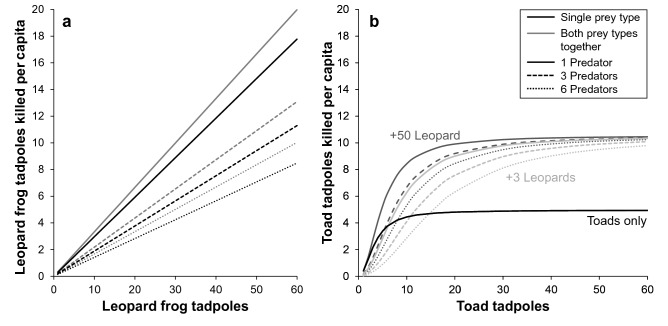
Functional response models for each prey type. Best fit functional response models are depicted for leopard frog (*Lithobates pipiens*) and toad (*Anaxyrus americanus*) tadpoles in the presence and absence of alternate prey. In cases where the best fit model had significant evidence of interference: solid lines indicate the per capita kill rate when with 1 predator, dashed lines indicate are the per capita kill rate when three predators were in the arena, and dotted lines indicates the kill rate with 6 predators. In (**b**) two sets of grey lines are presented to illustrate the effect of adding 3 (light grey) vs. 50 (dark grey) leopard frog tadpoles on the per capita kill rate of toad tadpoles. (**b**) has only one black line because when toad tadpoles were the only prey available the best-fit model was prey-dependent.

To evaluate the factors governing the pattern and magnitude of predation we experimentally manipulated prey density, the relative abundance of each prey type, and predator density in experimental arenas and recorded predator kill rate on each prey type. We used this data to fit functional response models which enabled us to examine differences between prey types (i.e., in terms of ‘prey availability’ (*α*), handling time (*h*), and the effect of interference (*m*)), as well as the effects that alternate prey might have on these metrics. As a first step we diagnosed the shape of the functional response (i.e., hyperbolic vs. sigmoidal). When fitting functional response models to the bootstrapped toad-only data sets, one or both of the hyperbolic and sigmoidal models failed to converge in only 7.6% of cases. In the remaining 92.4% of cases where both models converged the sigmoidal model was always a better fit (hyperbolic models: ΔAIC_c_ > 32.8). For the leopard frog-only data sets, one or both models failed to converge in 2% of cases. When both models converged, a hyperbolic model was supported in 92.2% of the cases (ΔAIC_c_ > 2), the sigmoidal model was the better fit in 4.6% of cases, and both models were equally supported in only 3.2% of cases (ΔAIC_c_ < 2). Conclusions based on models fitted using the raw data (i.e., *n* = 24) were consistent with this result; the sigmoidal model was supported for toad-only data (hyperbolic model: ΔAIC_c_ = 17.0), and the hyperbolic model was supported for the leopard-frog only data (sigmoidal model: ΔAIC_c_ = 6.2). Parameter estimates revealed important distinctions in the functional response for the two prey types: toad-only: *α* = 0.56 (95% CI: 0.23–1.57), *h* = 0.20 (0.17–0.24), and *m* = 0.08 (0.00–0.50); leopard frog-only: *α* = 0.30 (0.21–0.40), *h* = 0.00 (0.00–0.00), and *m* = 0.41 (0.17–0.62) (Fig. 1). Thus, in addition to differences in shape of the functional response, the kill rate of unpalatable prey was influenced by a substantial handling time (*h*), but not by predator interference (*m*), whereas the predator kill rate of palatable prey was influenced strongly by interference and handling time had relatively little influence (at the prey densities investigated).

Adding alternate prey substantially influenced the kill rate of toad tadpoles, but had little impact on the kill rate of leopard frog tadpoles (Table 2, Fig. 1). The best fit model for predation on toad tadpoles in the presence of leopard frog tadpoles was a sigmoidal Arditi-Akçakaya model modified to include *q* (i.e., where the presence of alternate prey reduces the effective number of predators feeding on the focal prey) (Table 2). In contrast, the best fit model for predation of leopard frog tadpoles was a hyperbolic Arditi-Akçakaya model where interference is not influenced by alternate prey (Table 2). Notably, with toad tadpoles as the focal prey the interference parameter increased dramatically from *m* = 0.08 (95% CI: 0.00–0.50) in the absence of alternate prey to *m* = 0.38 (0.09–0.70) when alternate prey were present. Importantly, when both prey types were offered simultaneously the effect of interference on the kill rate of unpalatable prey became equal to the effect of interference on the kill rate of palatable prey (*m* = 0.40, 95% CI: 0.25–0.51). In addition, the handling time of toad tadpoles in the presence of leopard frog tadpoles (*h* = 0.10, 95% CI: 0.05–0.14) was half that of the handling time in their absence (*h* = 0.20, 95% CI: 0.17–0.24).

**Table 2:** Fitted functional response models when predators were offered two prey types.

	ΔAICc	*w*	*α*	*h*	*m*	*q*	*x*
**Focal prey: Leopard frog tadpoles**							
**Arditi-Akçakaya (hyperbolic)**	**0.00**	**0.52**	**0.33 (0.28–0.40)**	**0.00 (0.00–0.01)**	**0.40 (0.25–0.51)**		
Arditi-Akçakaya (sigmoidal)	14.55	0.00	0.10 (0.05–0.21)	0.12 (0.09–0.14)	0.46 (0.26–0.68)		
Arditi-Akçakaya (hyperbolic) *x*	1.70	0.22	0.33 (0.28–0.39)	0.00 (0.00–0.01)	0.42 (0.26–0.62)		0.01 (0.00–0.04)
Arditi-Akçakaya (sigmoidal) *x*	16.73	0.00	0.10 (0.05–0.21)	0.12 (0.09–0.14)	0.46 (0.25–0.68)		0.00 (0.00–0.01)
Arditi-Akçakaya (hyperbolic) *q*	2.18	0.18	0.33 (0.28–0.39)	0.00 (0.00–0.01)	0.39 (0.25–0.51)	0.00 (0.00–0.01)	
Arditi-Akçakaya (sigmoidal) *q*	16.73	0.00	0.10 (0.05–0.21)	0.12 (0.09–0.14)	0.46 (0.25–0.68)	0.00 (0.00–0.01)	
Arditi-Akçakaya (hyperbolic) *q* + *x*	3.92	0.07	0.33 (0.28–0.40)	0.00 (0.00–0.01)	0.42 (0.26–0.63)	0.00 (0.00–0.01)	0.01 (0.00–0.05)
Arditi-Akçakaya (sigmoidal) *q* + *x*	18.95	0.00	0.10 (0.05–0.21)	0.12 (0.09–0.14)	0.46 (0.25–0.68)	0.00 (0.00–0.01)	0.00 (0.00–0.01)
**Focal prey: Toad tadpoles**							
Arditi-Akçakaya (hyperbolic)	10.16	0.00	0.22 (0.16–0.28)	0.00 (0.00–0.01)	0.26 (0.06–0.43)		
Arditi-Akçakaya (sigmoidal)	16.50	0.00	0.13 (0.05–0.42)	0.17 (0.14–0.21)	0.54 (0.23–0.87)		
Arditi-Akçakaya (hyperbolic) *x*	12.33	0.00	0.22 (0.16–0.28)	0.00 (0.00–0.01)	0.26 (0.06–0.43)		0.00 (0.00–0.01)
Arditi-Akçakaya (sigmoidal) *x*	18.68	0.00	0.13 (0.05–0.42)	0.17 (0.14–0.21)	0.54 (0.23–0.87)		0.00 (0.00–0.004)
Arditi-Akçakaya (hyperbolic) *q*	1.49	0.22	0.31 (0.22–0.50)	0.00 (0.00–0.01)	0.24 (0.05–0.41)	0.04 (0.01–0.14)	
**Arditi-Akçakaya (sigmoidal) ***q*	**0.00**	**0.47**	**0.14 (0.07–0.29)**	**0.10 (0.05–0.14)**	**0.38 (0.09–0.70)**	**0.07 (0.03–0.20)**	
Arditi-Akçakaya (hyperbolic) *q* + *x*	3.71	0.07	0.31 (0.22–0.50)	0.00 (0.00–0.01)	0.24 (0.05–1.11)	0.04 (0.01–0.14)	0.00 (0.00–0.00)
Arditi-Akçakaya (sigmoidal) *q* + *x*	1.47	0.23	0.14 (0.07–0.29)	0.10 (0.05–0.14)	0.38 (0.08–1.36)	0.07 (0.03–0.20)	0.00 (0.00–0.00)

Models that include *x* and *q* parameters were included to evaluate mechanisms through which alternate prey might influence the effect of interference on the kill rate of the focal prey. ΔAIC_c_ = delta Akaike information criterion corrected for small sample size, *w* = AIC_c_ weight, *α* = prey availability, *h* = handling time, *m* = mutual interference coefficient, *q* = degree to which alternate prey reduces the effective number of predators, *x* = the effect of alternate prey density on indirect predator interference. Values in parentheses represent 95% confidence intervals. Bold font indicates the best fit model for each focal prey type.

**Figure 2:**
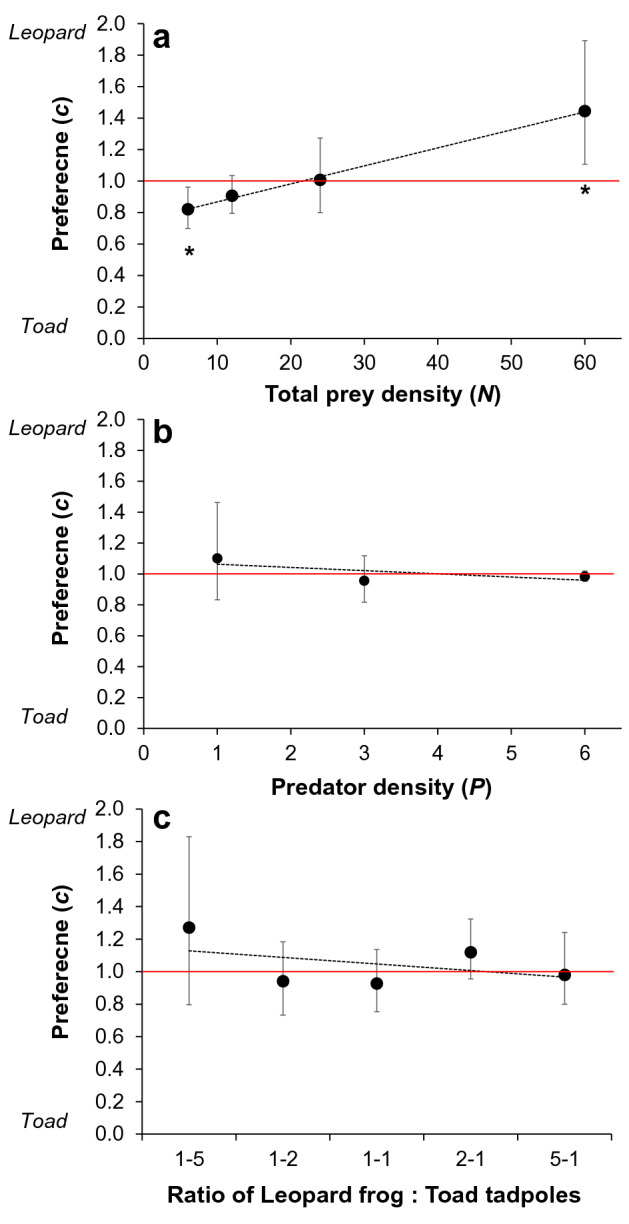
Strength of prey selection. Points indicate estimated prey selection coefficient values (*c*) ± 95% confidence intervals. Significant prey preference is indicated when the 95% confidence interval for the estimated value of *c* does not overlap with 1. Values < 1 indicate preference for toad tadpoles and values > 1 indicate preference for leopard frog tadpoles. (**a**) the effect of overall prey density on preference, (**b**) illustrates that predator density had no effect of prey selection, and (**c**) illustrates that selection was not influenced by the ratio of leopard frog : toad tadpoles. Solid red horizontal lines indicate no prey selection (*c* = 1), and asterisks indicate significant selective predation.

**Figure 3:**
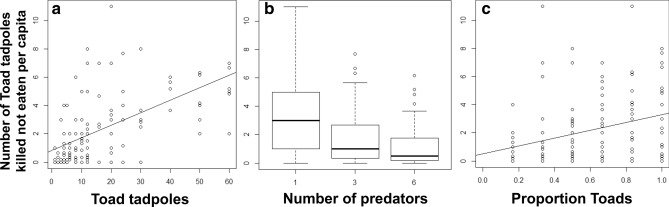
Predictors of wasteful killing. Number of toad tadpoles killed but not eaten per capita (i.e., wasteful killing), as influenced by toad tadpole density (**a**), predator density (**b**), and proportion of total available prey that were toad tadpoles (**c**).

Prey selection was significantly influenced by total prey density (Fig. 2), with a significant preference for toad tadpoles when total prey density was low (*c* = 0.82, 95% CI: 0.70–0.96), and a significant preference for leopard frog tadpoles when prey density was high (*c* = 1.44, 95% CI: 1.11–1.89). Preference was not influenced by predator density, or relative abundance of each prey type, however, there was more variation in preference in tanks with higher predator density and when the ratio of leopard frog: toad tadpoles was low (Fig. 2). Wasteful killing was negligible for leopard frog tadpoles (proportion of available killed but not eaten: median = 0, interquartile range = 0.05), but varied significantly with toad tadpoles (median = 0.34, interquartile range = 0.5; Fig. 3). The number of toad tadpoles killed but not eaten per capita decreased with predator density (*t*_138_ = −4.69, *p* < 0.001), increased with toad tadpole density (*t*_138_ = 7.27, *p* < 0.001), and increased (though not significantly) with proportion of toad tadpoles (*t*_138_ = 1.72, *p* = 0.088) (Fig. 3). This best fit model had an AIC_c_ weight of 0.51. The next best fit model (ΔAICc = 0.86) included only predator density and toad tadpole density, and had an AIC_c_ weight of 0.33. Remaining models were > 2 AICc from the best fit model (see Supplementary Table S3).

## Discussion

In our experiments, predators took longer to capture leopard frog tadpoles (palatable prey) and consumed a smaller proportion of each American toad tadpole (unpalatable prey), despite spending roughly the same amount of time consuming each prey type. In isolation from alternate prey, predation of the leopard frog tadpoles was limited by ‘prey availability’ (*α*) and predator interference (*m*), whereas predation on toad tadpoles was limited almost exclusively by handling time (*h*). The addition of unpalatable toad tadpoles did not impact the predator’s functional response to the palatable leopard frog tadpoles, but the addition of palatable leopard frog tadpoles dramatically altered the predator’s functional response to the unpalatable toad tadpole prey. Specifically, the presence of leopard frog tadpoles significantly decreased ‘prey availability’ (*α*) and handling time (*h*) of toad tadpoles and shifted the functional response from prey-dependent (*m* ≈ 0) to predator-dependent (*m* ≈ 0.38). Notably, this shift brought the level of interference in the toad tadpole functional response up to the same value observed in palatable leopard frog tadpoles. The effect of interference on the kill rate of toad tadpoles, however, was modulated by the density of palatable leopard frog tadpole prey such that increases in the number of palatable prey resulted in a greater number of toad tadpoles being killed. Concurrently, prey selection was influenced by total prey density, not the relative availability of either prey type, and shifted from a preference for unpalatable mobile prey (toad tadpoles) at low densities to a preference for relatively inactive palatable prey (leopard frog tadpoles) when prey density was high. Our results illustrate that the effects of alternate prey on the predator’s functional response depend on the identity of prey types involved, and that prey selection may be governed by distinct processes at low vs. high total prey density.

The effect of alternate prey on the per capita kill rate of the focal prey was not symmetrical. Adding alternate prey only influenced the kill rate of the focal prey type when alternate prey was a palatable prey type (i.e., leopard frog tadpoles). Interestingly, we found that increasing the density of palatable prey increased the rate at which unpalatable prey were killed. Our models suggest that palatable prey ‘distract’ foraging predators that would otherwise be engaged in interactions with other predators^12^, thereby freeing up concurrently foraging predators to capture and kill unpalatable prey. In addition, the presence of palatable alternate prey decreased the ‘prey availability’ (*α*) and decreased the handling time (*h*) of the unpalatable prey type. Given that an increase in one prey type indirectly reduced the survival of a competitor through effects on a shared predator, this clearly reflects a form of predator-mediated apparent competition^43,44^. More specifically, this is an example of “short-term” apparent competition because it is mediated through changes in predator foraging behaviour^45,46^, instead of through numerical changes in predator density^44^.

Mechanistically, we propose that predators shifted from a sit-and-wait foraging strategy when palatable prey were absent to active search when they were present. Although we did not collect predator behaviour data here, we have previously documented analogous shifts in dragonfly nymph foraging behaviour associated variation in prey density^35^. Active search would increase encounter rate with the palatable, but relatively immobile, leopard frog tadpoles while concurrently increasing encounter with the unpalatable toad tadpoles and conspecific predators. Indeed, the observed increase in prey consumption and interference (*m*) when leopard frog tadpoles were added as alternate prey is consistent with this explanation. Our observation of negligible interference when only the highly mobile unpalatable toad tadpoles were available is also consistent with dragonfly nymphs adopting an energetically efficient sit-and-wait hunting strategy which concurrently reduced their encounter rate with conspecifics. Importantly, there were no indicators of shift in foraging mode on palatable prey upon the addition of unpalatable prey. Our results are therefore broadly consistent with the general prediction from optimal diet theory that predator foraging behaviour should be governed primarily by the availability of preferred prey.

Although dragonfly nymphs can actively distinguish between palatable and unpalatable tadpoles^47^, we found no evidence of frequency-dependent switching behaviour (sensu^48,49^). Instead, prey selection shifted from a significant preference for mobile unpalatable prey at low total prey density to a significant preference for less active palatable prey at high total prey density. A shift in prey selection resulting from changes in total prey density (i.e., “rank switching”) has been observed in other generalist predators^33^, but its general importance has been largely overlooked. Our profitability trials indicate that toad tadpoles are easier for dragonfly nymphs to obtain (lower time to capture), but that a greater proportion of each leopard frog tadpole captured was consumed. The switch from a preference for easy-to-capture prey when overall prey density is low to a preference for the more palatable prey type when overall prey density is high, as documented here, is consistent with previous theoretical expectations for rank switching^33,34^. Our results therefore support the hypothesis that predation by generalist predators on mobile prey will tend to be governed by prey vulnerability at low total prey density, but by predator preference when total prey density is high.

In contrast to the traditional predictions of optimal diet theory, we found that: (1) predators generalized their diet at intermediate total prey densities and specialised on distinct prey types at very high vs. very low total prey density, (2) the decision to specialize depended on total prey density rather than the density of a single preferred prey type, and (3) the switch from generalist to specialist was gradual. The density-dependent shifts in prey preference observed here may indicate that at low prey density the marginal benefit from eating easy-to-capture unpalatable prey (toad tadpoles) was greater than that from rejection and waiting to encounter the harder-to-capture palatable prey (leopard frog tadpoles). Active foraging acts to equalize the encounter rate between active and inactive prey perhaps enabling predators to be more selective, but active search may also be energetically inefficient when the overall reward rate is low (e.g., at low total prey density^22^). Interestingly, a substantive amount of unpalatable prey mortality was caused by ‘wasteful killing’. Following prey capture, predators can decide how much time to invest in consuming prey (e.g.^50–52^), and modulating the extent of partial consumption of captured prey items is another means to optimize energy intake^53,54^. Toxins in toad tadpoles appear to be concentrated in the skin^55,56^ and other parts likely remain palatable and nourishing^57^. Thus, taste-rejection and partial consumption of toxic prey may help predators balance conflicting demands of maximizing ingestion rate while limiting toxin intake. We found that partial consumption is typical of odonate nymphs feeding on toad tadpoles, but not leopard frog tadpoles, indicating that wasteful killing was modulated by prey palatability.

The patterns of predation observed here are broadly in line with the expectations of aposematism, where predators learn to avoid unprofitable or toxic prey^58^. When palatable prey are rare or absent, predators are expected to learn quickly that prey are unpalatable^59^ but must still make strategic decisions to consume toxic or unpalatable prey to avoid starvation^60,61^. When palatable and unpalatable types are presented together, predators may require additional sampling before they can discriminate among prey types and avoid aposematic prey. In addition, Sherratt^59^ showed that as unprofitable prey become more common, they should be sampled more before they are rejected completely (i.e., sampling of unfamiliar prey is density-dependent), but also that there should be an upper asymptote to the number of toxic prey that sampled prior to complete rejection. The sigmoidal functional response we observed for toad tadpoles is consistent with a density-dependent optimal sampling strategy for unfamiliar chemically-defended prey constrained by some upper asymptote, followed by permanent avoidance of that prey type (Fig. 1). Yet, while previous work has indicated that predation on unpalatable prey should decline as palatable prey increase in availability (e.g.^62,63^), we found that unpalatable toad tadpoles suffered higher rates of predation when palatable leopard frog tadpoles were simultaneously available, in part because of the effect palatable alternate prey had on predator interference.

When foraging on toxic prey, predators may reach a toxin load threshold which temporarily limits further consumption of toxic prey (e.g.^60,61^). Jeschke^42^ suggested that predators which consume chemically-defended prey experience an extended ‘digestive pause’, which can increase the handling time of toxic prey. Consistent with this, unpalatable prey had a significantly longer handling time in our functional response experiment, and several lines of evidence suggest that consumption of toad tadpoles may have been limited by toxin load. Specifically, consumption time was qualitatively longer for toad tadpoles in the profitability trials despite predators consuming a smaller proportion of their carcasses (Table 1), and handling time for toad tadpoles decreased in trials where palatable prey were available (Fig. 1). Moreover, wasteful killing per capita declined at higher predator densities, but increased with both the abundance of toad tadpoles, and the proportion of available prey that were toad tadpoles (Fig. 3). We note that the negligible handing time for palatable prey reflects the relative importance of ‘prey availability’ and predator interference in governing the per capita kill rate on relatively inactive prey types, as well as the short duration of our foraging trial, rather than a functional absence of handling time constraints (see^64^).

Our experimental work reveals a number of important patterns that are not intuitive based on classic ecological theory. Specifically, we found no evidence of frequency-dependent prey switching (sensu^9^), instead prey switching depended on the total number of prey available. Second, increasing the availability of palatable prey increased the kill rate of unpalatable prey via a form of short-term apparent competition, and third, increasing the availability of palatable prey induced predator-dependence in the kill rate of unpalatable prey where the kill rate was otherwise prey-dependent. Each of these points reinforces our growing understanding of the ecological complexity involved in natural systems and is broadly consistent with recent theoretical developments. Excitingly, there appears to be much left for us to discover in the context of predator–prey interactions, and we encourage theoreticians, empiricists, and field biologists to increase their collaborative efforts to further unravel the mechanisms and processes involved.

## Material and methods

### Animal collection & husbandry

Leopard frog (*Lithobates pipiens*) and American toad (*Anaxyrus americanus*) egg masses were collected from ponds around Peterborough, Ontario (44° 22′ N 78° 03′ W) and reared to Gosner stage 25. Tadpoles were fed ad libitum on a diet of ground algae discs. Late instar Aeshnidae dragonfly nymphs were collected by dip-netting ponds, including those where the amphibian egg masses were collected, and were housed individually in small plastic dishes filled with 400 ml of aged ozonated river water. Nymphs were offered tadpoles from both species prior to use in our trials.

### Ethics statement

To minimise the impact of egg collection on wild populations we collected only partial egg mass from the field. Both anuran species used in this experiment have large brood sizes, and survival from the egg to adult stage is low for most anurans, including the species we examined. Survival and growth are also density-dependent^65–67^, further minimizing the effects of egg collection on wild populations. High statistical power is required to diagnose differences in the shape of the functional response^68^. Recent work has further indicated that the quantification of predator dependence has suffered from systematic bias due to lack of sufficient replication^69^. We leveraged the hidden replication built into the factorial design of our functional response experiment in order to minimize the replication necessary to achieve sufficient power to test our hypotheses. Specifically, we employed a 4 × 3 × 7 design (total prey density × predator density × focal:alternate prey ratio) which ensured that our analyses covered the broad range of prey densities, prey:predator ratios, and alternate prey availabilities necessary to parameterize our models. Bootstrapping further enabled us to reduce the number of replicates needed to determine the functional response shape and ensure the reliability of our parameter estimates in our single prey type trials. The sample size employed here was therefore the minimum that would enable us to test our hypotheses. All procedures were approved by the Trent University Animal Care Committee, and all experiments were performed in accordance with relevant guidelines and regulations including ASAB/ABS and ARRIVE Guidelines (https://arriveguidelines.org).

### Profitability trials

In optimal diet theory, ‘profitability’ has traditionally been defined as the energetic reward per unit of handing time (e.g.^8,32^). Our use of the term follows this definition. These trials were an attempt to explore potential differences in profitability between the prey types, given that logistical constraints prevented us from being able to make detailed behavioural observations of the predators during the functional response experiment (see below). Twenty-four hours before each trail we standardized predator hunger by feeding each nymph 3 leopard frog tadpoles. During trials, nymphs were placed individually in circular arenas (diameter = 11 cm) with a single rock located in the center to act as a perch. Following a 15 min acclimation period they were presented with a single tadpole (leopard frog or toad). We recorded the time to first strike, number of strikes to successful capture, time to capture, and consumption time. Once the nymph had finished consuming a tadpole, or had dropped the remaining carcass, they were presented with a second tadpole and the same data were recorded. Tadpoles were weighed prior to use in a trial, as was the mass of any carcass remaining after it had been dropped, enabling us to quantify the proportion of each tadpole consumed. Strike success was calculated as 1 / number of strikes. A total of 36 trials were run, split evenly among four tadpole sequences (leopard frog-leopard frog, leopard frog-toad, toad-toad, toad-leopard frog, i.e., *n* = 9 replicates per sequence). Individual nymphs were only used in a single trial and were not used in the functional response experiment described below. Leopard frog tadpoles used in these trials weighed an average (± SE) of 0.071 ± 0.005 g and toad tadpoles weighed 0.070 ± 0.005 g. Note that prey used in this part of the study were larger than those used in the functional response experiment below due to the availability of prey at the time when this aspect of the work was conducted.

### Functional response experiment set-up

During this experiment the wet mass of leopard frog tadpoles was 0.029 ± 0.0013 g (mean ± SE) and the wet mass of American toad tadpoles was 0.022 ± 0.0010 g. Predation trials took place in tanks measuring 50.8 × 25.4 cm filled to 10 cm with water. Visual cues from adjacent tanks were blocked by affixing wax paper to the back and sides, and tank bottoms were lined with plastic mesh enabling nymphs to move and feed naturally. Each tank received a total of 6, 12, 24 or 60 tadpoles, with leopard frog and toad tadpoles combined according to one of seven leopard frog : toad tadpole ratios (1:0, 5:1, 2:1, 1:1, 1:2, 1:5, 0:1). We then added 1, 3, or 6 late-instar Aeshnidae dragonfly nymphs to each tank enclosing them in separate inverted bottomless plastic cups to prevent feeding and contact among nymphs until the start of trials. Once all animals were arranged in tanks, they were left undisturbed to acclimate for 15 min. To standardize hunger, nymphs were given 3 leopard frog tadpoles 12 h before each trial and tadpoles were fed ad libitum for 24 h prior to the trials. Following acclimation, nymphs were released and allowed to feed for 3 h. The number of each prey type remaining in each tank was enumerated visually at 20-min intervals. To ensure accurate differentiation between species, leopard frog tadpoles were dyed with 0.02 g ⁄ l neutral red immediately before trials [^70^, see Supplementary Material]. On 3 occasions, one nymph captured and killed another in which case we immediately replaced the killed nymph to maintain constant predator density^30^. Predators were removed after 3 h. We enumerated the number of tadpoles remaining alive and the number of tadpoles killed but not fully consumed (i.e., > 50% of carcass remaining) separately for each prey species. Due to the overt variation in prey and predator density among treatments, the experimenter could not be blinded to the experimental treatment. For each prey species we calculated the number of tadpoles killed as the number at the start of the trial—the number remaining alive. In total, 168 predation trials were completed over 4 days (i.e., *n* = 2 replicates per frog: toad ratio × total prey density × predator density). We used a stratified-random approach to determine the combination of tanks that were run each day, using computer based random order generator.

### Analyses

Preliminary analyses of behavioural data from the staged profitability trials in small arenas revealed that the presentation sequence did not have a significant influence. We therefore examined differences between the two prey species via Mann–Whitney U Tests using data from the first prey offered. A single-sample t-test was used to test whether the amount of each prey item consumed differed between prey types because nymphs invariably consumed 100% of leopard frog tadpoles.

Using the data collected in the functional response experiment, we fit the Arditi-Akçakaya functional response models^23,25^ modified to account for depletion (Eq. 2 derived by^29^) and compared the fit of hyperbolic vs. sigmoidal versions using a model selection approach based on ΔAIC_c_^71^.

1 $${N}_{e}={N}_{0} (1-\mathrm{exp}(-\alpha \left({P}^{1-m}\right))*T+\alpha {P}^{-m}h{N}_{e})$$

2 $${N}_{e}= \frac{{P}^{2m}+b{{N}_{0}}^{2}h+ b{N}_{0}PT \pm \sqrt{-4{b}^{2}{{N}_{0}}^{3}PhT+({P}^{2m}+b{{N}_{0}}^{2}h+ b{N}_{0}PT{)}^{2}}}{2b{N}_{0}h}$$

where *N*_*e*_ is the number of prey killed, *N*_*0*_ is the initial number of the focal prey available, *P* is the number of predators, *T* is the duration of the trial (in hours), *α* is ‘prey availability’ (analogous to, but mathematically distinct from, the ‘*attack rate*’ parameter in Holling-type functional response models), *h* is handling time, and *m* is the mutual interference coefficient. In the sigmoidal model (Eq. 2) *α* increases as a linear function of prey density (*b* = *α N*_*0*_). A larger *α* reflects cases where the rate at which prey are made available to predator population is greater, *h* is a measure of the time it takes to pursue, subdue, consume, and digest a single prey item^25^. The mutual interference coefficient (*m*) indicates the degree to which predator density depresses an individual predator’s kill rate, usually ranging between 0 (no effect or predator density) and 1 (strong effects of predator density). We predicted that interference would be stronger when predators are feeding on the preferred prey type.

In some cases, all prey were consumed prior to the end of the 3 h feeding trial which can bias estimates of the functional response parameters when fitting models that account for prey depletion. Specifically, in 51 trials all leopard frog tadpoles had been consumed, and in 74 trials all of the toad tadpoles had been consumed or killed. We therefore fit functional response models to the number of tadpoles killed after 1 h, which eliminated most cases where all prey were consumed. In single-prey trials this left only 4 cases where all toad tadpoles were killed, and no cases where all leopard frog tadpoles were killed within 1 h. In multi-prey trials, there was only a single case where all prey were killed within 1 h. Separate models were fit for each prey type when presented in isolation from alternate prey (i.e., leopard frog only, American toad only), however, because only *n* = 24 data points were available in these cases we used bootstrapping to ensure that parameters for single-prey models were estimated accurately. Specifically, prior to fitting the models we resampled our raw data to generate 2000 new data sets with n = 120 data points separately for each scenario. Hyperbolic and sigmoidal Arditi-Akçakaya functional response models were fit to each of these 2000 data sets, then parameter estimates were extracted and AIC_c_ values calculated for each model. Mean values from parameter estimate distributions were used to calculate ‘prey availability’ (*α*), handling time (*h*), and mutual interference coefficient (*m*), and the 95% confidence intervals were calculated as the 2.5% and 97.5% quantile of the parameter estimate distributions. Results from this approach produced qualitatively similar results to fitting functional response models to the raw *n* = 24 data sets (see Supplementary Material).

Next, we fit Arditi-Akçakaya functional response models to the kill rate data for each prey type when alternate prey were available. Here we had *n* = 120 data points for each prey type, and we similarly compared the fit of hyperbolic and sigmoidal versions of the model using AIC_c_. To evaluate how the addition of alternate prey influenced per capita kill rate of the focal prey type, we compared parameter estimates from the best fit multi-prey models against the best fit single-prey models. In addition, we examined modified Arditi-Akçakaya models which enabled us to evaluate mechanisms through which alternate prey might impact predator interference. Specifically, Tschanz et al.^12^ recognised that presence of alternate prey might influence predator interference through two non-mutually exclusive mechanisms: (1) by reducing the effective number of predators (i.e., by distracting predators from foraging on their primary prey), (2) by reducing the magnitude of indirect interference (e.g., by slowing depletion of “easy-to-find” prey)^12^. Following Tschanz et al.^12^, the former mechanism can be tested by substituting *P*/(*qN*_*2*_ + 1) for *P*, where *q* represents the degree to which alternate prey reduce the effective number of predators and *N*_*2*_ represents the density of alternate prey. The latter mechanism can be tested by substituting *m*/(*xN*_*2*_ + 1) for *m*, where *x* represents the degree to which alternate prey (*N*_*2*_) reduce the effect of interference on the per capita kill rate of *N*_*1*_. We used nonlinear regression to fit hyperbolic and sigmoidal models with either, both, and neither of these two modifications, first assuming leopard frog tadpoles as the primary prey and toad tadpoles as the alternate prey then again with toad tadpoles as the primary prey. Best fit models were determined using ΔAIC_c_.

We evaluated prey selection by fitting Eq. (3) to the number of each prey type killed and estimating *c*^9^.

3 $$P=cF/(1-F+cF)$$

where *F* represents the proportion of prey offered and *P* is the proportion of prey consumed. A value of *c* = 1 indicates no selection, *c* > 1 indicates preference for leopard frog tadpoles, and *c* < 1 indicates preference for toad tadpoles; *c* cannot be < 0. Significant preference is indicated when the 95% confidence interval of *c* does not overlap with 1. To determine the effects of total prey density, predator density, and leopard frog : toad tadpole ratio on prey selection *c* was fit separately for each total prey density, each predator density, and each leopard frog : toad tadpole ratio.

The correlates of wasteful killing (i.e., prey killed but not consumed) were identified by comparing general linear models using a model selection approach. The number of the focal prey type killed but not eaten (i.e., < 50% of the carcass consumed) per capita was the response variable, and predictors included number of the focal prey offered, total number of prey offered, number of alternate prey offered, predator density, proportion of the focal prey offered, total prey offered/number of predators (i.e., *N*/*P*), leopard frog tadpoles offered/number of predators, and toads offered/number of predators. Variables were scaled in the analyses and the response variable was transformed to meet the assumption of normally distributed residuals. Models with correlated predictors were excluded from the candidate set of models, and the full set of candidate models is listed in the Supplementary Material. All analyses were conducted in R version 3.6.1^72^.

## Supplementary Information

Supplementary Information

## Data Availability

The datasets generated during and/or analysed during the current study are available from the corresponding author on reasonable request. The data will also be archived in a public repository.

## References

[1] Elton CS (1927). Animal Ecology.

[2] Curio E (1976). The Ethology of Predation.

[3] Stephens DW, Brown JS, Ydenberg RC (2007). Foraging: Behavior and Ecology.

[4] Holling CS (1959). The components of predation as revealed by a study of small mammal predation of the European pine sawfly. Can. Entomol..

[5] Hassell MP, Varley GC (1969). New inductive population model for insect parasites and its bearing on biological control. Nature.

[6] Beddington JR (1975). Mutual interference between parasites or predators and its effect on searching efficiency. J. Anim. Ecol..

[7] DeAngelis DL, Goldstein RA, O’Neill RV (1975). A model for tropic interaction. Ecology.

[8] Stephens DW, Krebs JR (1986). Foraging Theory.

[9] Murdoch WW, Avery S, Smyth MEB (1975). Switching in predatory fish. Ecology.

[10] Akre BG, Johnson DM (1979). Switching and sigmoid functional response curves by damselfly naiads with alternative prey available. J. Anim. Ecol..

[11] Benhadi-Marín J, Pereira JA, Sousa JP, Santos SAP (2019). Functional responses of three guilds of spiders: comparing single- and multiprey approaches. Ann. Appl. Biol..

[12] Tschanz B, Bersier LF, Bacher S (2007). Functional responses: a question of alternative prey and predator density. Ecology.

[13] Sih A, Christensen B (2001). Optimal diet theory: when does it work, and when and why does it fail?. Anim. Behav..

[14] Nakano S, Fausch KD, Kitano S (1999). Flexible niche partitioning via a foraging mode shift: a proposed mechanism for coexistence in stream-dwelling charrs. J. Anim. Ecol..

[15] Kullberg C (1995). Strategy of the Pygmy Owl while hunting avian and mammalian prey. Ornis Fenn..

[16] Oaten A, Murdoch WW (1975). Switching, functional response, and stability in predator-prey systems. Am. Nat..

[17] Abrams PA (1999). The adaptive dynamics of consumer choice. Am. Nat..

[18] Abrams PA, Kawecki TJ (1999). Adaptive host preference and the dynamics of host–parasitoid interactions. Theor. Popul. Biol..

[19] van Baleen M, Krivan V, van Rijn P, Sabelis M (2001). Alternative food, switching predators and the persistence of predator-prey systems. Am. Nat..

[20] Formanowicz DR, Bradley PJ (1987). Fluctuations in prey density: effects on the foraging tactics of scolopendrid centipedes. Anim. Behav..

[21] Hirvonen H (1999). Shifts in foraging tactics of larval damselflies: effects of prey density. Oikos.

[22] Hassell MP (1978). The Dynamics of Arthropod Predator–Prey Systems.

[23] Arditi R, Akçakaya HR (1990). Underestimation of mutual interference of predators. Oecologia.

[24] Abrams PA, Ginzburg LR (2000). The nature of predation: prey dependent, ratio dependent or neither?. Trends Ecol. Evol..

[25] Arditi R, Ginzburg LR (2012). How Species Interact: Altering the Standard View of Trophic Ecology.

[26] Chan K (2017). Improving the assessment of predator functional responses by considering alternate prey and predator interactions. Ecology.

[27] Tyutyunov YV, Titova LI (2020). From Lotka-Volterra to Arditi-Ginzbug: 90 years of evolving trophic functions. Biol. Bull. Rev..

[28] Novak M, Stouffer DB (2020). Systematic bias in studies of consumer functional responses. Ecol. Lett..

[29] Schenk D, Bersier LF, Bacher S (2005). An experimental test of the nature of predation: neither prey- nor ratio-dependent. J. Anim. Ecol..

[30] Hossie TJ, Murray DL (2016). Spatial arrangement of prey affects the shape of ratio-dependent functional responses in strongly antagonistic predators. Ecology.

[31] Pulliam HR (1974). On the theory of optimal diets. Am. Nat..

[32] Charnov EL (1976). Optimal foraging: attack strategy of a mantid. Am. Nat..

[33] Baudrot V, Perasso A, Fritsch C, Giraudoux P, Raoul F (2016). The adaptation of generalist predators’ diet in a multi-prey context: insights from new functional responses. Ecology.

[34] Palma L, Beja P, Pais M, Da Fonseca LC (2006). Why do raptors take domestic prey? The case of Bonelli’s eagles and pigeons. J. Appl. Ecol..

[35] Hossie TJ, Murray DL (2010). You can’t run but you can hide: refuge use in frog tadpoles elicits density-dependent predation by dragonfly larvae. Oecologia.

[36] Hossie TJ, Murray DL (2012). Assessing behavioural and morphological responses of frog tadpoles to temporal variability in predation risk. J. Zool..

[37] Relyea RA (2001). Morphological and behavioral plasticity of larval anurans in response to different predators. Ecology.

[38] Hossie TJ, Landolt K, Murray DL (2017). Determinants and co-expression of anti-predator responses in amphibian tadpoles: a meta-analysis. Oikos.

[39] Relyea RA (2001). The relationship between predation risk and antipredator responses in larval anurans. Ecology.

[40] Shine R (2010). The ecological impact of invasive cane toads (*Bufo marinus*) in Australia. Quart. Rev. Biol..

[41] Üveges B, Fera G, Móricz ÁM, Krüzselyi D, Bókony V, Hettyey A (2017). Age- and environment-dependent changes in chemical defences of larval and post-metamorphic toads. BMC Evol. Biol..

[42] Jeschke JM (2006). Density-dependent effect of prey defences and predator offences. J. Theor. Biol..

[43] Holt RD (1977). Predation, apparent competition, and the structure of prey communities. Theor. Popul. Biol..

[44] Chaneton EJ, Bonsall MB (2000). Enemy-mediated apparent competition: empirical patterns and the evidence. Oikos.

[45] Holt RD, Kotler BP (1987). Short-term apparent competition. Am. Nat..

[46] Abrams PA (1993). Effect of increased productivity on the abundances of trophic levels. Am. Nat..

[47] Jara FG, Perotti MG (2009). Toad tadpole responses to predator risk: ontogenetic change between constitutive and inducible defenses. J. Herpetol..

[48] Murdoch WW (1969). Switching in general predators: experiments on predator specificity and stability of prey populations. Ecol. Monogr..

[49] Chesson PL (1984). Variable predators and switching behavior. Theor. Popul. Biol..

[50] Gende SM, Quinn TP, Willson MF (2001). Consumption choice by bears feeding on salmon. Oecologia.

[51] Skelhorn J, Rowe C (2006). Predator avoidance learning of prey with secreted or stored defences and the evolution of insect defences. Anim. Behav..

[52] Vucetich JA, Vucetich LM, Peterson RO (2012). The causes and consequences of partial prey consumption by wolves preying on moose. Behav. Ecol. Sociobiol..

[53] Sih A (1980). Optimal foraging: partial consumption of prey. Am. Nat..

[54] Lucas JR, Grafen A (1985). Partial prey consumption by ambush predators. Theor. Popul. Biol..

[55] Halliday DC (2009). Cane toad toxicity: an assessment of extracts from early developmental stages and adult tissues using MDCK cell culture. Toxicon.

[56] Toledo RC, Jared C (1995). Cutaneous granular glands and amphibian venoms. Comp. Biochem. Physiol. Part A Mol. Integr. Physiol..

[57] Parrott ML, Doody JS, McHenry C, Clulow S (2019). Eat your heart out: choice and handling of novel toxic prey by predatory water rats. Aust. Mammal..

[58] Ruxton GD, Allen WL, Sherratt TN, Speed MP (2018). Avoiding Attack: The Evolutionary Ecology of Crypsis, Aposematism, and Mimicry.

[59] Sherratt TN (2011). The optimal strategy for sampling unfamiliar prey. Evolution.

[60] Skelhorn J, Rowe C (2007). Predators' toxin burdens influence their strategic decisions to eat toxic prey. Curr. Biol..

[61] Barnett CA, Skelhorn J, Bateson M, Rowe C (2012). Educated predators make strategic decisions to eat defended prey according to their toxin content. Behav. Ecol..

[62] Nonacs P (1985). Foraging in a dynamic mimicry complex. Am. Nat..

[63] Sherratt TN (2003). State-dependent risk-taking by predators in systems with defended prey. Oikos.

[64] Jeschke JM, Kopp M, Tollrian R (2004). Consumer-food systems: why type I functional responses are exclusive to filter feeders. Biol. Rev..

[65] Wilbur HM (1977). Density-dependent aspects of growth and metamorphosis in *Bufo americanus*. Ecology.

[66] Loman J (2004). Density regulation in tadpoles of *Rana temporaria*: a full pond experiment. Ecology.

[67] Yagi KT, Green DM (2016). Mechanisms of denity-dependent growth and survival in tadpoles of Fowler’s Toad, *Anaxyrus fowleri*: volume vs. abundance. Copeia.

[68] Marshal JP, Boutin S (1999). Power analysis of wolf-moose functional responses. J. Wild. Manag..

[69] Novak M, Stouffer DB (2020). Systematic bias of consumer functional responses. Ecol. Lett..

[70] Hossie TJ, Murray DL (2011). Effects of structural refuge and density on foraging behaviour and mortality of hungry tadpoles subject to predation risk. Ethology.

[71] Burnham KP, Anderson DR (2002). Model Selection and Multimodel Inference: A Practical Information-Theoretic Approach.

[72] R Core Team. R: A language and environment for statistical computing. R Foundation for Statistical Computing, Vienna, Austria. https://www.R-project.org/ (2019).

