# Supplementary material for: Increasing availability of palatable prey induces predator-dependence and increases predation on unpalatable prey
Source: Sci Rep. 2021 Mar 24;11:6763. doi: 10.1038/s41598-021-86080-x (PMC7991668; doi:10.1038/s41598-021-86080-x)
Supplement: Supplementary file 1 — Supplementary Information [file 41598_2021_86080_MOESM1_ESM.docx]

**Supplementary Material for:**

**Increasing availability of palatable prey induces predator-dependence and increases predation on unpalatable prey**

Thomas J. Hossie^1*^, Kevin Chan^1^, Dennis L. Murray^1^

¹Department of Biology, Trent University, 2140 East Bank Drive, Peterborough, ON K9J 7B8

*Corresponding author email: [thossie@trentu.ca](mailto:thossie@trentu.ca) Phone: 705-748-1011 x6346

Co-author emails: [kevinchan4@trentu.ca](mailto:kevinchan4@trentu.ca), [dennismurray@trentu.ca](mailto:dennismurray@trentu.ca)

**Table S1**: Comparison of parameter estimates generated from bootstrapping vs. fitting models directly to the *n* = 24 empirically-derived data points for predation on toad tadpoles by dragonfly nymphs in the absence of alternate prey. From the bootstrapping procedure, one or both of the models failed to converge in 153 cases (7.65%), and in all cases were both models converged the sigmoidal Arditi-Akçakaya (AA) was the better fit (all ΔAICc > 32.8).

| Estimates based on bootstrapped data | | | | |  | | Fit using only original n = 24 data points | | | | | |  |
| --- | --- | --- | --- | --- | --- | --- | --- | --- | --- | --- | --- | --- | --- |
|  | | | | |  | |  | | | | | |  |
| **Toad - Hyperbolic AA** | | |  | |  | | **Toad - Hyperbolic AA** | | | |  |  |  |
| % convergence: 99.90% | | | | |  | | AICc: 168.783 | | | |  |  |  |
|  | | | | |  | |  | | | |  |  |  |
|  | **Median** | **Mean** | **LCI** | **UCI** | |  | |  | **Est.** | **SE** | **t** | **P** | |
| ***a*** | 0.144 | 0.147 | 0.113 | 0.194 |  | | ***a*** | | 0.134 | 0.095 | 1.422 | 0.170 |  |
| ***h*** | 0.000 | 0.000 | 0.000 | 0.000 |  | | ***h*** | | 0.000 | 0.075 | 0.000 | 1.000 |  |
| ***m*** | 0.023 | 0.077 | 0.000 | 0.321 |  | | ***m*** | | 0.020 | 0.344 | 0.058 | 0.954 |  |
|  |  |  |  |  |  | |  | |  |  |  |  |  |
| **Toad - Sigmoidal AA** | | |  | |  | | **Toad - Sigmoidal AA** | | | |  |  |  |
| % convergence: 92.45% | | |  | |  | | AICc: 151.697 | | |  |  |  |  |
|  | | |  | |  | |  | | |  |  |  |  |
|  | **Median** | **Mean** | **LCI** | **UCI** |  | |  | | **Est.** | **SE** | **t** | **P** |  |
| ***a*** | 0.452 | 0.560 | 0.228 | 1.568 |  | | ***a*** | | 0.395 | 1.272 | 0.310 | 0.759 |  |
| ***h*** | 0.203 | 0.204 | 0.171 | 0.243 |  | | ***h*** | | 0.203 | 0.021 | 9.752 | 0.000 |  |
| ***m*** | 0.000 | 0.083 | 0.000 | 0.502 |  | | ***m*** | | 0.000 | 1.166 | 0.000 | 1.000 |  |

**Table S2**: Comparison of parameter estimates generated from bootstrapping vs. fitting models directly to the *n* = 24 empirically-derived data points for predation on leopard frog tadpoles by dragonfly nymphs in the absence of alternate prey. From the bootstrapping procedure, one or both models failed to converge in 41 cases (2.05%), and in cases were both models converged the hyperbolic Arditi-Akçakaya (AA) model was supported (ΔAICc > 2) in 1807 cases (92.2%), both models were equally supported (ΔAICc < 2) in 62 cases (3.2%), and a sigmoidal AA fit better (ΔAICc > 2) in 90 cases (4.6%).

| Estimates based on bootstrapped data | | | | |  | Fit using only original n = 24 data points | | | | |
| --- | --- | --- | --- | --- | --- | --- | --- | --- | --- | --- |
|  |  |  |  |  |  |  | | | | |
| **Leopard - Hyperbolic AA** | | | | |  | **Leopard - Hyperbolic AA** | | | | |
| % convergence: 100% | | | | |  | AICc: 147.202 | | | |  |
|  | | | | |  |  | | | |  |
|  | **Median** | **Mean** | **LCI** | **UCI** |  |  | **Est.** | **SE** | **t** | **P** |
| ***a*** | 0.296 | 0.299 | 0.211 | 0.402 |  | ***a*** | 0.297 | 0.095 | 3.124 | 0.005 |
| ***h*** | 0.000 | 0.000 | 0.000 | 0.000 |  | ***h*** | 0.000 | 0.020 | 0.000 | 1.000 |
| ***m*** | 0.414 | 0.409 | 0.173 | 0.617 |  | ***m*** | 0.417 | 0.165 | 2.520 | 0.020 |
|  |  |  |  |  |  |  |  |  |  |  |
| **Leopard - Sigmoidal AA** | | | | |  | **Leopard - Sigmoidal AA** | | | | |
| % convergence: 97.95% | | | | |  | AICc: 153.385 | | | |  |
|  | | |  | |  |  | | |  |  |
|  | **Median** | **Mean** | **LCI** | **UCI** |  |  | **Est.** | **SE** | **t** | **P** |
| ***a*** | 0.133 | 0.155 | 0.046 | 0.396 |  | ***a*** | 0.143 | 0.174 | 0.822 | 0.420 |
| ***h*** | 0.103 | 0.104 | 0.057 | 0.154 |  | ***h*** | 0.103 | 0.019 | 5.437 | 0.000 |
| ***m*** | 0.685 | 0.688 | 0.432 | 0.969 |  | ***m*** | 0.724 | 0.350 | 2.070 | 0.051 |

**Table S3**: AIC_c_ table for candidate models seeking to explain wasteful killing of American toad (*Anyxrus americanus*) tadpoles. The predictor variable was the number of toad tadpoles killed, but with < 50% of the carcass consumed / number of foraging predators (i.e., wasteful killing *per capita*). N/P indicates the ratio of total prey : predators, T/P indicates the ratio of toad tadpoles : predators, L/P indicates the ratio of leopard frog tadpoles : predators.

| **Model** | **AICc** | **ΔAICc** | **w** | **Adjusted R²** |
| --- | --- | --- | --- | --- |
| **PredatorDensity + Toad + propToad** | **330.31** | **0.00** | **0.505** | 0.4269 |
| PredatorDensity + Toad | 331.16 | 0.86 | 0.329 | 0.4189 |
| PredatorDensity + Leopard + Toad | 332.57 | 2.26 | 0.163 | 0.4177 |
| propToad + TotalPrey | 342.20 | 11.90 | 0.001 | 0.3719 |
| Leopard + T/P + propToad | 342.39 | 12.08 | 0.001 |  |
| Leopard + Toad + propToad | 343.96 | 13.65 | 0.001 |  |
| propToad + N/P | 345.12 | 14.81 | 0 |  |
| N/P + propToad | 345.12 | 14.81 | 0 |  |
| Toad + L/P | 345.47 | 15.16 | 0 |  |
| PredatorDensity + Leopard + propToad | 346.30 | 15.99 | 0 |  |
| Toad + propToad | 349.12 | 18.81 | 0 |  |
| Toad | 349.70 | 19.39 | 0 |  |
| Leopard + Toad | 351.22 | 20.91 | 0 |  |
| PredatorDensity + TotalPrey | 353.37 | 23.06 | 0 |  |
| propToad + T/P | 355.19 | 24.89 | 0 |  |
| propToad + L/P | 361.07 | 30.76 | 0 |  |
| T/P | 361.68 | 31.37 | 0 |  |
| Leopard + propToad | 362.66 | 32.35 | 0 |  |
| Leopard + T/P | 362.68 | 32.38 | 0 |  |
| TotalPrey | 369.05 | 38.74 | 0 |  |
| N/P | 371.55 | 41.25 | 0 |  |
| PredatorDensity + propToad | 374.22 | 43.91 | 0 |  |
| propToad | 386.91 | 56.61 | 0 |  |
| PredatorDensity | 395.47 | 65.17 | 0 |  |
| PredatorDensity + Leopard | 395.88 | 65.58 | 0 |  |
| L/P | 402.40 | 72.10 | 0 |  |
| Leopard | 406.65 | 76.35 | 0 |  |

**Table S4**: AIC_c_ table for candidate models seeking to explain wasteful killing of Northern Leopard frog (*Lithobates pipiens*) tadpoles. The predictor variable was the number of toad tadpoles killed, but with < 50% of the carcass consumed / number of foraging predators (i.e., wasteful killing *per capita*). N/P indicates the ratio of total prey : predators, T/P indicates the ratio of toad tadpoles : predators, L/P indicates the ratio of leopard frog tadpoles : predators.

| **Model** | **AICc** | **ΔAICc** | **w** | **Adjusted R²** |
| --- | --- | --- | --- | --- |
| **L/P** | **324.57** | **0.00** | **0.487** | 0.4409 |
| propToad + L/P | 326.00 | 1.43 | 0.238 | 0.4396 |
| Toad + L/P | 326.66 | 2.09 | 0.171 |  |
| PredatorDensity + Leopard | 328.72 | 4.15 | 0.061 |  |
| PredatorDensity + Leopard + propToad | 330.81 | 6.24 | 0.021 |  |
| PredatorDensity + Leopard + Toad | 330.85 | 6.28 | 0.021 |  |
| Leopard | 342.39 | 17.82 | 0 |  |
| Leopard + T/P | 343.13 | 18.57 | 0 |  |
| Leopard + propToad | 344.46 | 19.90 | 0 |  |
| Leopard + Toad | 344.48 | 19.91 | 0 |  |
| Leopard + T/P + propToad | 345.09 | 20.52 | 0 |  |
| Leopard + Toad + propToad | 346.42 | 21.85 | 0 |  |
| propToad + TotalPrey | 359.13 | 34.56 | 0 |  |
| PredatorDensity + TotalPrey | 360.33 | 35.76 | 0 |  |
| TotalPrey | 371.23 | 46.66 | 0 |  |
| PredatorDensity + Toad + propToad | 380.58 | 56.02 | 0 |  |
| PredatorDensity + propToad | 388.79 | 64.23 | 0 |  |
| propToad + T/P | 388.84 | 64.27 | 0 |  |
| Toad + propToad | 389.80 | 65.23 | 0 |  |
| propToad + N/P | 395.83 | 71.26 | 0 |  |
| N/P + propToad | 395.83 | 71.26 | 0 |  |
| propToad | 396.92 | 72.35 | 0 |  |
| PredatorDensity | 398.73 | 74.17 | 0 |  |
| PredatorDensity + Toad | 400.51 | 75.94 | 0 |  |
| N/P | 405.17 | 80.60 | 0 |  |
| T/P | 406.25 | 81.68 | 0 |  |
| Toad | 407.87 | 83.31 | 0 |  |

**Use of red dye to distinguish tadpole species:**

Use of the red dye exclusively on leopard frog tadpoles was in part a logistic constraint. Toad tadpoles are so dark in their colouration that even after applying the red dye we were unable to reliably distinguish dyed toad tadpoles from leopard frog tadpoles. Red colouration does not appear to be a warning colouration in tadpoles. In fact, some tadpoles develop pigmented tails (with black and/or red colouration) in the presence of predatory dragonfly nymphs (e.g., [1]), perhaps to deflect attacks toward the tail and away from the body (e.g., [2]). Hossie and Murray [3] used red dye to distinguish between hungry vs. well-fed American bullfrog tadpoles (*Lithobates catesbeiana*). We contend that the differences in kill rate between leopard frog and toad tadpoles to be large relative to any effect of red dye. It is not clear whether dragonfly nymphs can see red, however it is well known that dragonfly nymphs rely primary on motion cues to detect their prey. Our results show clearly that, in the absence of handling time constraints, attack rate was higher on the toad tadpoles than leopard frog tadpoles (Figure 1), further suggesting that any modest effect of dye was overwhelmed by species differences in activity.

**Profitability:**

In optimal diet theory, ‘profitability’ has traditionally been defined as the energetic reward per unit of handing time (e.g., [4-5]). Our use of the term follows this definition. The circular arenas used in these trials were small enough to keep prey within a detectable distance. Therefore, to estimate profitability for each prey type we can sum tCapture (i.e., pre-capture pursuit time) and tConsume (i.e., manipulation time after capture) from our ‘profitability trials’ to estimate handling time. Because prey were size-matched in these trials we can use proportion of each prey item consumed as a proxy for energy value per prey item. The values used below are the species-level averages presented in Table 1 in the main text.

For leopard frog tadpoles this was:

$$\frac{1}{(447.72 s+525.17 s)}=0.00103$$

For toad tadpoles this was:

$$\frac{0.79}{(189.67 s+574.72 s)}=0.00103$$

These calculations provide staggeringly similar profitability estimates, and profitability calculations are similarly close if the overall species-level averages from the first and second prey offered are used instead of the averages from the first prey offered only. Thus, despite leopard frog tadpoles providing more food per prey captured, the two prey types appear to have similar profitability in terms of energy gained per unit of handling time (based on these trials). We note that prey used in these profitability trials were larger than those used in the functional response experiment below, so these estimates will not translate directly to that experiment.

Data from functional response experiments can be used to obtain analogous parameter estimates for handing time (*h*) from fitting functional response models. Prey in the functional response trails were smaller than in the profitability trials, and our models indicated that handling time was negligible for leopard frog tadpoles, but was about 0.1 h (360 s) for toad tadpoles when both prey types were available (Table 2). We note that the negligible handing time for leopard frog tadpoles prey reflects the relative importance of ‘prey availability’ (*α*) and predator interference (*m*) in governing the *per capita* kill rate on this relatively inactive prey type, as well as the short duration of our foraging trial, rather than a functional absence of handling time constraints (see [6]). That said, the negligible handling time for leopard frog tadpoles here prevents us from being able to make calculations of profitability using the data from this experiment to compare prey types. During this experiment there was a substantial amount of “wasteful killing” (i.e., those prey killed where <50% of carcass was consumed), but this was almost completely restricted to the toad tadpoles (see main text). We contend that even if the profitability of each of these prey types was also equal during the functional response experiment (i.e., as was the case during the profitability trials), that leopard frog tadpoles would still likely be the preferred prey type due to the unpalatability and skin toxins in toad tadpoles. Future research that estimates handling time from behavioural observations during functional response trails would be well suited to continue testing hypotheses about optimal foraging when chemically-defended prey are available.

**References:**

1. Touchon, J. C. & Warkentin, K. M. Fish and dragonfly nymph predators induce opposite shifts in color and morphology of tadpoles. *Oikos* **117**, 634-640 (2008).
2. Van Buskirk, J., Aschwanden, J., Buckelmüller, I., Reolon, S., & Rüttiman, S. Bold tail coloration protects tadpoles from dragonfly strikes. *Copeia* **2004**, 599–602 (2004).
3. Hossie, T. J. & Murray D. L. Effects of structural refuge and density on foraging behaviour and mortality of hungry tadpoles subject to predation risk. *Ethology* **117**, 777-785 (2011).
4. Stephens, D. W. & Krebs, J. R. Foraging theory. (Princeton University Press, 1986).
5. Charnov, E. L. Optimal foraging: attack strategy of a mantid. *Am. Nat.* **110**, 141–151 (1976).
6. Jeschke, J. M., Kopp, M. & Tollrian, R. Consumer-food systems: why type I functional responses are exclusive to filter feeders. Biol. Rev. 79, 337–349 (2004).
